# Assessing for autism in adult psychiatry

**DOI:** 10.1192/bjo.2023.542

**Published:** 2023-08-08

**Authors:** Samuel Tromans, Pushpal Desarkar

**Affiliations:** Department of Population Health Sciences, University of Leicester, Leicester, UK; and Adult Learning Disability Service, Leicestershire Partnership NHS Trust, Leicester, UK; Azrieli Adult Neurodevelopmental Centre, Centre for Addiction and Mental Health, Toronto, Ontario, Canada; and Department of Psychiatry, University of Toronto, Toronto, Ontario, Canada

**Keywords:** Autism, adults, psychiatry, co-occurring conditions, functioning

## Abstract

This editorial discusses a study by Nyrenius and colleagues in which they investigated rates of co-occurring psychiatric conditions and functioning in a population of adults referred to a Swedish psychiatric out-patient clinic, comparing those meeting DSM-5 diagnostic criteria for autism with their non-autistic peers.

Autism (referred to as autism spectrum disorder in DSM-5) is a neurodevelopmental condition characterised by persistent impairments in social communication and social interaction, in addition to ‘restricted, repetitive patterns of behaviour, interests, or activities’.^1^ Additionally, autistic people can have special talents and abilities, with Happé^2^ citing examples such as enhanced memory for detail and musical and other artistic talents. However, not all autistic people have special abilities, and some parents have reported that a focus on such skills in the media can be unhelpful.^2^ The reported prevalence of autism in the general population is around 1%.^1^ Furthermore, autistic people have been previously reported to have heightened rates of a wide range of mental health conditions compared with their non-autistic peers.^3^

In a study population of adults referred to a Swedish psychiatric out-patient clinic, Nyrenius and colleagues^4^ compared autistic adults with their non-autistic peers in relation to the presence of co-occurring mental health conditions, as well as psychosocial functioning.

## Methodology

A significant strength of the study is that the authors adopted an active case-sampling approach, whereby they identified the autistic group through diagnostic assessment. This contrasts with a passive case-sampling approach, whereby the autistic and non-autistic groups are defined according to presence or absence of a pre-existing clinical diagnosis of autism on their medical records.^5^ The advantage of active case sampling is that it supports identification of autistic people whose autism previously remained unrecognised through routine clinical care. With passive case sampling, only diagnosed autistic people are identified; previous research demonstrates that many autistic adults are undiagnosed.^6^ Furthermore, the characteristics of previously diagnosed autistic people, such as rates of co-occurring mental health conditions, may not necessarily be representative of all autistic people (i.e. undiagnosed plus diagnosed).

Nyrenius and colleagues conducted detailed clinical assessments to determine whether patients met diagnostic criteria for autism according to DSM-5.^1^ However, they did cite difficulties in obtaining a parent-reported developmental history, with only 34% (*n =* 31) of the study population having returned these forms. This is a frequent challenge when attempting to obtain a developmental history from the parents of adult patients, as their parents may have died or they may be estranged from their parents, for example.^7^

However, it is important to acknowledge that the ‘merged-ASD group’ comprised not just adults meeting the full DSM-5 diagnostic criteria for autism spectrum disorder (*n =* 52), but also those meeting criteria for ‘subthreshold ASD’ (*n* = 11). Subthreshold ASD was defined as ‘meeting two out of three necessary A-criteria and at least two B-criteria of ASD according to the DSM-5’. Thus, the autistic group was not solely comprised of adults fully satisfying DSM-5 diagnostic criteria for autism spectrum disorder.

## Mental health conditions

The authors report using an interview (the Mini-International Neuropsychiatric Interview version 7.0.1 with an ADHD supplement^8^) and two tests (the Alcohol Use Disorders Identification Test^9^ and the Drug Use Disorders Identification Test^10^) to identify presence of mental health conditions in both the autistic and non-autistic groups, as well as identifying tic disorder via observation of tics during the interview assessment and/or participant self-report. However, such an approach also requires appreciation by assessing researchers that such conditions can present differently in autistic persons.

Interestingly, for most of the mental health conditions reported, there was no significant difference in rates between the autistic and non-autistic participant groups, with significant differences (Bonferroni-corrected alpha-level *P* = 0.0045) reported for only non-autistic neurodevelopmental disorders (attention-deficit hyperactivity disorder and/or tic disorders) and anxiety disorders. (The *P*-value was corrected because multiple comparisons were being made, which increased the risk of type 1 error, i.e. false positives.^11^) This result may in part be related to the relatively small size of the study population, with the autistic and non-autistic groups comprising 63 and 27 participants respectively. However, it may also reflect the study population being psychiatric out-patients; although increased rates of mental health conditions in autistic people have been widely observed,^3^ many such findings are from general population samples, and it is possible that rates among autistic and non-autistic adults utilising mental health services are more similar.

## Psychosocial functioning

Global Assessment of Functioning (GAF)^12^ scores were found to be significantly lower (*P* < 0.01) in the autistic group compared with their non-autistic peers. However, 26.7% of the variability in GAF scores in the autistic group was attributable to co-occurring psychiatric conditions. This suggests that much of the functional impairment reported by the autistic group is not directly related to their autism.

However, although the GAF is a widely used measure, its reliability and validity when used in adult psychiatric out-patient populations has been brought into question in previous studies.^13^

## Conclusions

As the authors point out, a limitation of the study is the low participation rate; one approach that could have been taken to mitigate this is to have additionally reported the demographic characteristics of all patients attending the specialist psychiatric clinic between 1 January 2019 and 31 December 2020, to indicate how representative the study population was of the wider patient group from which it was drawn.

The authors very reasonably recommend careful assessment for presence of co-occurring mental health conditions in autistic patients, as treatment of these will optimise functioning, as well as likely overall well-being. However, to achieve this, it is essential that clinicians have a good understanding of autism in order to appreciate how mental health conditions can present differently in this patient group, as well as possible symptoms of mental health conditions potentially overlapping with autistic features.^7^

The authors also recommend that autism should be ‘ruled out’ in adult psychiatric patients by taking a developmental history. The National Institute for Health and Care Excellence (NICE) guidelines for autism in adults^14^ suggest use of the 10-item version of the Autism Spectrum Quotient (AQ-10)^15^ to support identification of autism in adults without moderate or severe intellectual disability. They advise that a full diagnostic autism assessment should be offered to patients scoring ≥6 on the AQ-10 or if autism is suspected based on clinical judgement and any informant history.^14^ However, further research is required to establish effective, efficient means of checking for presence/absence of autism in this patient population.

Overall, this study is a valuable addition to the current research evidence base relating to the characteristics of autistic adults accessing out-patient psychiatric services, particularly owing to its active case-sampling method and thorough diagnostic assessment of those participants considered to have a high likelihood of being autistic. In light of the single-site nature of the study and relatively small sample size, such findings need to be synthesised with those of similar studies in order to draw firmer conclusions that can inform policy.

## Data Availability

Data availability is not applicable to this article as no new data were created or analysed in this study.
